# Performance and antioxidant traits of broiler chickens fed with diets containing rapeseed or flaxseed oil and optimized quercetin

**DOI:** 10.1038/s41598-023-41282-3

**Published:** 2023-08-28

**Authors:** Kamil Sierżant, Eliza Piksa, Damian Konkol, Kamila Lewandowska, Muhammad Umair Asghar

**Affiliations:** 1 Department of Animal Nutrition and Feed Science, The Faculty of Biology and Animal Science, Wrocław University of Environmental and Life Sciences, Chełmońskiego St. 38C, 51-630 Wrocław, Poland; 2 Department of Environmental Hygiene and Animal Welfare, The Faculty of Biology and Animal Science, Wrocław University of Environmental and Life Sciences, Chełmońskiego St. 38C, 51-630 Wrocław, Poland

**Keywords:** Animal physiology, Biochemistry

## Abstract

This study evaluated the effect of quercetin (Q) added to feed mixtures, at concentrations directly optimized for the peroxidability of dietary rapeseed (RO) and flaxseed oil (FLO), on performance and selected biomarkers of oxidative stress of broiler chickens. Ninety-six one-day-old Ross 308 broiler chicken males were randomly assigned to four groups (six replicates per treatment, four birds per cage, n = 24 per group): Group RO received diets containing rapeseed oil (RO) and group FLO received diets containing flaxseed oil (FLO); Group RO_Q and group FLO_Q received these same diets containing RO or FLO oils, supplemented with optimized quercetin (Q). Blood, pectoral muscles, and liver samples of chickens were collected after 35 days to determine: (1) the global indicators of antioxidant capacity: ferric reducing antioxidant power (FRAP), antiradical activity (DPPH^·^/ABTS^·+^), total antioxidant status (TAS), and glutathione peroxidase (GSH-Px); (2) the activity of the antioxidant enzymes catalase (CAT) and superoxide dismutase (SOD); and (3) the concentration of malondialdehyde (MDA). Data showed that the FLO diet did not affect the final performance parameters in relation to RO, but the optimized Q tended to improve the total body weight gain and the final body weight of broiler chickens (*P* = 0.10). The antioxidant traces analyzed in the blood (GSH-Px), plasma (FRAP, ABTS^·**+**^, DPPH^·^, TAS), serum (DPPH^·^), and pectoral muscles (SOD, CAT) of chickens were not altered by either Oil or Q factor. FLO supplementation increased MDA content in the liver of chickens (*P* < 0.05) and increased liver CAT activity, which was not improved by optimized Q. Meanwhile, the Oil × Q interaction suggests that optimized Q could reduce the liver burden and negative effects of oxidized lipid by-products associated with FLO diets. Our results indicate that optimizing the addition of natural polyphenols to feed may be a valuable alternative to the application of polyphenolic antioxidants in animal nutrition, allowing for an economical use of the antioxidant additives when customized to the peroxidability of fat sources, which is line to the conception of sustainable development covering ‘The European Green Deal’ and ‘Farm to Fork Strategy’.

## Introduction

Quercetin (pentahydroxyflavone) is a flavonoid compound that belongs to flavonols. It can be found in the skins of edible fruits such as grapes, apples and blueberries, in vegetables such as red onions, capers, cruciferous vegetables, shallots, green peppers and asparagus, and in many seeds, flowers, nuts, leaves and bark^1–4^. When ingested, quercetin has properties that are potentially beneficial to both human and animal health and it is accumulated and maintained in the organism more effectively than other flavonoids^5^. Recent studies have shown that quercetin has strong antioxidant and antibacterial properties^6–8^. In vitro studies in animal and human models have shown that it can reduce inflammatory immune response—often by modulating the production of pro- and anti-inflammatory molecules by cells of the innate and acquired immune systems (e.g. macrophages and T cells), in response to stimuli that increase inflammatory processes^9,10^. Quercetin has also been shown to exert anticancer properties, and act as supportive treatment of diabetes, obesity, and neurological diseases^11^.

The exceptionally high antioxidant properties of quercetin have recently attracted the interest of researchers for its use as a feed additive in animal nutrition. This is because antioxidant mediators reduce the peroxidation of feed lipids, thus improving the organoleptic properties and nutritional value of the eggs or meat obtained from animals—and it extends the shelf life of these products^12,13^. The most important mechanism in the antioxidant action of quercetin is an ability to scavenge free radicals. Through this mechanism, quercetin can directly inhibit the oxidation of low-density lipoproteins and protect erythrocytes from the harmful effects of some environmental factors^14,15^. Additionally, the flat structure of the quercetin molecule^16^ facilitates its penetration deep into the hydrophobic core of lipid membranes^17,18^, which may enhance its antioxidant efficiency up to 150-fold^17^. As a strong chelator, quercetin also inhibits the production of hydroxyl radicals (OH^·^) through the chelation of intracellular iron^19^.

Currently, studies are being conducted to assess the possibility of using quercetin in animal nutrition—especially in poultry. The main focus of research upon quercetin is directed on improving the performance of broiler chickens and improving the quality and oxidative stability of poultry meat^20^. However, the studies have not definitively shown quercetin to have a positive or negative effect on the performance of broiler chickens. The results of Goliomytis et al*.*^3^ showed no improvement in production parameters (body weight, feed intake) of chickens receiving 0.5 and 1 g of quercetin/kg of feed. These authors also found that chickens receiving quercetin had a significantly higher feed conversion ratio (FCR). On the other hand, Abdel-Latif et al*.*^21^ found that chickens fed with diets enriched with 200 and 400 ppm of quercetin had better growth. In a study by Wang et al*.*^22^, quercetin was used as an alternative to antibiotics, that shown the chickens which were fed with diets supplemented with quercetin were found to have significantly lower number of pathogenic bacteria, such as *Pseudomonas aeruginosa*, *Salmonella enterica* serotype *Typhimurium*, *Staphylococcus aureus* and *Escherichia coli*. Furthermore, a significantly higher number of probiotic bacteria from the genera *Lactobacillus* spp. and *Bifidobacterium* spp. was found in the cecum. Hager-Theodorides et al*.*^23^ proved that using dietary quercetin as an antioxidant could potentially enhance the humoral immune response of broilers, by increasing the production of primary IgY antibodies. Quercetin has also been used in the nutrition of laying hens. Liu et al*.*^24^ showed that the dietary addition of quercetin at levels of 0.2 and 0.4 g/kg of feed can significantly increase egg laying and reduce FCR. This study also indicated that quercetin can improve egg quality.

Despite the promising results obtained from multiple studies, no standardization for the use of quercetin and other polyphenolic additives in poultry nutrition has been developed. This lack of a clear standardization may be contributing to the inconsistency in the results obtained from different nutrition trials. Moreover, studies have shown that dietary polyphenols with high antioxidant properties may exert prooxidant activity under certain conditions, which can stimulate oxidative damage of cellular structures, including DNA, lipids and proteins^25^, or have a detrimental effects on peroxidability of the meat^26,27^. Because the concentration of polyphenolic compounds and other antioxidants plays an important role in determining their antioxidative and prooxidative action^26^, therefore, this ‘dual nature’ of phenolics can be attributed with Paracelsus statement: “All things are poison, and nothing is without poison; the dosage alone makes it so a thing is not a poison.”, which is recently known as ‘hormesis’^28^. In case of quercetin, the side effects of this flavonol could be related with its prooxidant properties, cancerogenic activity or mitochondrial toxicity^29,30^. However, the recent data confirmed that 14-weeks supplementation of approx. 31% of the median lethal dose (LD50 for mice = 160 mg/kg of BW)^31^ to diet of mice has no negative effects on their body composition, organ function, behavior and metabolism^32^. Notwithstanding, the high dose of quercetin (40.000 ppm; 1900 mg/kg of BW/day) administered to the diet of rats showed their carcinogenic activity in the kidney, but the growth of benign tumors of the renal tubule epithelium was observed only in the male rats, and after a long term period (2 years) of the supplementation^30^.

Considering the above, the aim of this study was to evaluate the performance of broiler chickens and the oxidative stability of poultry tissues, under the influence of optimized quercetin (Q) added to feeds containing plant oils. Two oils were included in this study: rapeseed oil (RO), which is relatively stable in terms of peroxidation, and flaxseed oil (FLO), which is extremely prone to oxidation, containing approximately 50% n − 3 fatty acids (FA). The presented results are part of a broader study aimed at obtaining poultry meat with an increased proportion of n − 3 FA, but without negative changes in the oxidative stability of the meat, which was presented in previous study by Sierżant et al*.*^33^. In this research the approximate average concentration of quercetin consumed by the chickens ranged from 11 to 42 mg/kg of BW (the maximum peak concentration of quercetin was approximately 17–65 mg/kg of BW), depending on the age of chickens and the applied diet.

## Methods

### Animal ethics

According to Polish law (Act of 15.01.2015 on the Protection of Animals Used for Scientific and Educational Purposes), the ethical approval for this experiment was not required because the experiment was carried out under standard production conditions, therefore, the birds were not exposed to excessive pain, suffering and stress. All procedures on animals were carried out in compliance with European Union regulations^34^. The maximum bird density was lower than 16 kg/m^2^ which was in accordance to the requirements of the European Council Directive 2007/43/EC^35^. The experimental protocol was consulted and approved by the Advisory Team for the Welfare of Animals of The Faculty of Biology and Animal Science of Wrocław University of Environmental and Life Sciences (Decision no. 1/2020). This study was carried out in compliance with ARRIVE's guidelines.

### Experimental design and diets

This experiment lasted 35 days and was performed at the Wroclaw University of Environmental and Life Sciences experimental facilities, as described previously^33^. A total of 96 one-day-old Ross 308 broiler chicken males (46.6 ± 1.4 g) were randomly divided into four dietary treatment groups, with each treatment group of 24 birds further divided into 6 replicates with 4 birds per cage. The ambient temperature was progressively reduced from the initial 32 °C (1st day) to a final 25 °C (35th day) and the lighting regime included 18 h of light and 6 h of darkness. All broilers had free access to feed and to water via nipples.

The broiler chicks were fed with iso-energetic and iso-protein starter (day 1–14), grower (day 15–28) and finisher (day 29–35) diets given in mash form to the four treatment groups: RO (diets supplemented with rapeseed oil), FLO (diets supplemented with flaxseed oil), RO_Q (diets supplemented with RO plus the optimized dose of quercetin), FLO_Q (diets supplemented with FLO plus the optimized dose of quercetin), the composition of which is presented in Table 1. All diets were served in a mash form, and their formulation was adjusted to meet the nutrient requirements of Ross 308 chickens^36^. Metabolisable energy (ME) was calculated based on the results of chemical analyses of feed components according to Official Methods of Analysis^37^ and The Polish Nutritional Recommendations and Nutritional Value of Poultry Feed guidelines^38^, as described in our previous work^33^.

**Table 1:** Composition of the experimental starter and grower diets fed to broiler chickens^33^.

Items	Starter		Grower		Finisher	
	RO/RO_Q	FLO/FLO_Q	RO/RO_Q	FLO/FLO_Q	RO/RO_Q	FLO/FLO_Q
Maize, %	29.51	29.51	23.05	23.05	34.05	34.05
Wheat, %	19.52	19.52	30.32	30.32	21.10	21.10
Soybean meal, %	38.88	38.88	34.39	34.39	32.93	32.93
Rapeseed or flaxseed oil, %	6.19	6.19	6.92	6.92	7.58	7.58
Sodium bicarbonate, %	0.49	0.49	0.49	0.49	0.50	0.50
Phosphate 1 Ca, %	1.40	1.40	1.18	1.18	1.02	1.02
Chalk, %	1.07	1.07	0.67	0.67	0.24	0.24
L-Lysine, %	0.36	0.36	0.38	0.38	0.21	0.21
L-Methionine, %	0.31	0.31	0.31	0.31	0.20	0.20
L-Threonin, %	0.27	0.27	0.30	0.30	0.18	0.18
Vitamin/mineral, % premix*/**	2.00	2.00	2.00	2.00	2.00	2.00
Quercetin (Q)***, mg/kg of feed	0/66	0/243	0/72	0/269	0/79	0/296
Metabolizable Energy [MJ/kg of feed] and essential nutrients of experimental diets [g/kg of feed]						
ME	12.60	12.59	13.00	12.99	13.40	13.38
Dry matter	912.89	912.89	914.44	914.44	911.13	911.13
Crude protein	220.00	220.00	210.00	210.00	200.00	200.00
Crude fiber	25.86	25.86	25.37	25.37	25.34	25.34
Ca	10.50	10.50	9.00	9.00	7.50	7.50
P available	4.50	4.50	4.00	4.00	3.50	3.50
Na [g/kg]	1.60	1.60	1.60	1.60	1.60	1.60
Lysine**—**total	12.00	12.00	11.50	11.50	9.50	9.50
Methionine**—**total	5.50	5.50	5.20	5.20	4.30	4.30
Threonin**—**total	8.00	8.00	8.00	8.00	6.60	6.60
n − 6/n − 3 FA ratio in RO and FLO oil	2.71	0.29	2.71	0.29	2.71	0.29
Selected fatty acid traces determined in feed mixtures**** [g/kg of feed]^33^						
C18:1 (oleic acid)	4.14/4.55	1.77/1.91	4.86/4.81	2.16/1.98	5.15/5.74	2.56/2.15
C18:2 (linoleic acid)	1.66/1.69	1.57/1.69	1.72/1.87	1.87/1.71	1.85/1.85	2.07/1.85
C18:3 (α-linolenic acid)	0.37/0.35	2.74/3.08	0.40/0.47	3.35/3.33	0.41/0.42	3.65/3.34
n-6/n − 3 FA ratio determined in feed mixtures	4.48/4.82	0.57/0.54	4.30/3.97	0.55/0.51	4.51/4.40	0.56/0.55

RO, diets with rapeseed oil (RO); RO_Q, diets with RO supplemented with the optimized dose of quercetin (Q); FLO, diets with flaxseed oil (FLO); FLO_Q, diets with FLO supplemented with the optimized dose of Q.

*Chemical composition of the starter premix per 1 kg of diet: CaCO_3_**—**2.4 g; P available**—**2.4 g; Mn**—**80 mg; J**—**1.2 mg; Zn**—**100 mg; Fe**—**90 mg; Cu**—**20 mg; Se**—**350 µg; retinol**—**12,500 IU; cholecalciferol**—**5000 UI; α-tocopherol**—**82.5 mg; menadione**—**4 mg; thiamin**—**3 mg; riboflavin**—**9 mg; pyridoxine hydrochloride**—**6 mg; cyanocobalamin**—**40 µg; pantothenic acid**—**18 mg; biotin**—**300 µg; nicotinic acid**—**60 mg; folic acid**—**3 mg; choline chloride**—**402.6 mg; Lys**—**2.34 g; Met**—**2.2 g; Thr**—**0.32 g; phytase**—**600 OUT (phytase activity unit); xylanase**—**8000 EPU (endo-pentosanase units); coccidiostat**—**salinomycin; Proviox (equivalent of vitamin E)**—**75 mg; propyl gallate (the manufacturer did not provide the quantity of the antioxidant).

**Chemical composition of the finisher premix per 1 kg of diet: CaCO_3_**—**3.4 g; P available**—**1.7 g; Mn**—**70 mg; J**—**1 mg; Zn**—**80 mg; Fe**—**50 mg; Cu**—**15 mg; Se**—**300 µg; retinol**—**10,000 IU; cholecalciferol**—**3500 UI; α-tocopherol**—**33 mg; menadione**—**2.5 mg; thiamin**—**2 mg; riboflavin**—**5 mg; pyridoxine hydrochloride**—**3 mg; cyanocobalamin**—**20 µg; pantothenic acid**—**12 mg; biotin**—**200 µg; nicotinic acid**—**40 mg; folic acid**—**1.5 mg; choline chloride**—**200 mg; Lys**—**2.2 g; Met**—**1.4 g; Thr**—**0.34 g; phytase**—**600 OUT; xylanase**—**8000 EPU; Proviox (equivalent of vitamin E)**—**50 mg; propyl gallate (the manufacturer did not provide the quantity of the used antioxidant).

***Sum of ‘Component A and ‘Component B’; ’Component A’: concentration of quercetin used for protection of oils in feed mixtures, based on the calculated IC50 for Q and concentration of oils. The ‘Component A’ was applied for RO_Q starter, grower, and finisher mixtures at concentrations: 58, 65 and 71.5 mg/kg of feed, respectively; for FLO_Q starter, grower, and finisher mixtures at concentrations: 235, 262 and 288 mg/kg of feed. ‘Component B’: concentration of Q used both RO_Q and FLO_Q mixtures at concentrations approx. 7.79 (starter), 7.24 (grower) and 8.16 (finisher) mg/kg of kg feed.

****Determined according to PN-EN ISO 12,966–2^39^ and PN-EN ISO 12,966–4^40^.

The optimal concentration of Q added to the feed mixtures was quantified by determining the effective amount that inhibits the peroxidation process induced in the RO and FLO oils by approximately 50% (IC50): 0.943 g of Q/kg for RO and 3.797 g of Q/kg of FLO^33^. This target addition of Q per 1 kg of feed was applied according to the concentration of oils in the starter, grower, and finisher diet formulations (‘Component A’) and was further enhanced with an additional quercetin buffer (‘Component B’). The proportion of Q in the additional ‘Component B’ was equal to 50% of the IC50 values quantified for the RO oil and was applied to all feed mixtures containing quercetin (RO_Q and FLO_Q diets) based on fat concentration determined in feed components. The final concentrations of Q applied to the feed mixtures (sum of ‘Component A’ and ‘Component B’, mg/kg of feed) are given in Table 1. The application of Q to the prepared feed recipes was performed by mixing an equal amount of quercetin calculated for each diet with a dedicated amount of oil (RO or FLO). Subsequently, the oils or oils containing quercetin were pre-mixed with a small amount of the basal diet (starter, grower or finisher) and further mixed with rest of components.

### Performance indices

The body weight (BW) of each bird was determined at the beginning of the experiment (day 1), and then on the 14th, 28th and 35th day. Feed intake per cage, including leftovers, was measured at 2-week intervals, on the 1st day (beginning of starter period), on the 14th day (end of starter and beginning of grower period), on the 28th day (end of grower and beginning of finisher period) and on the 35th day (at the end of the experiment). The feed conversion ratio (FCR) was calculated by dividing the total weight of consumed feed (for starter, grower, finisher, and the total period of the experiment), by the net growth of the chickens (the weight of birds determined for each period, minus their starting weight).

### Blood and tissue sample collection

On the 35th day, two randomly selected birds from each replicate were slaughtered in accordance with the procedure described in Annex IV to Directive 2010/63/EU^41^. Blood samples were collected in EDTA tubes, heparin tubes, or Eppendorf tubes during postmortem exsanguination of the birds. Blood designated for obtaining plasma (EDTA) was kept on ice until centrifugation (3000×*g*, 4 °C, 15 min) (centrifuge MPW-352R, Poland) and then stored at − 80 °C for analyses (Arctico ULUF 450-2M, Denmark). Blood serum (centrifuged at 3500×*g*, 20 °C, 15 min) for total antioxidant status (TAS) and whole blood for determining glutathione peroxidase (GSH-Px) was stored at − 20 °C. Liver tissue and *pectoralis superficialis* muscle samples (approx. 4 cm distal part of the muscle) were collected no later than 10 min after death. The tissues were cut into small pieces (10 × 5 mm), immediately frozen in a liquid nitrogen, and then stored at − 80 °C (Arctico ULUF 450-2M, Denmark).

### Antioxidant status in serum and blood plasma, and glutathione peroxidase in whole blood

Total antioxidant activity in blood plasma or serum was estimated either by a 2,2-diphenyl-1-picrylhydrazyl (DPPH^·^) free radical assay in a 10 mM, 7.4 pH sodium phosphate buffer^42^, or by using 2,2ʹ-azino-bis(3-ethylbenzothiazoline-6-sulfonic acid) in an ABTS^·+^ cation-radical protocol^43^ and ferric reducing antioxidant ability of plasma (FRAP)^44^ protocols. The absorbance of DPPH^·^ at a wavelength of 516 nm was read with a Halo DB-20 Double Beam Spectrophotometer (Dynamica Scientific Ltd., United Kingdom), while for ABTS^·+^ (λ = 730 nm) and FRAP (λ = 595 nm) a Biotek EPOCH2 microplate reader was used (BioTek Instruments, Inc., USA). The assays were performed in triplicates and the results were expressed as the Trolox equivalent of antioxidant capacity (TEAC) of the plasma or blood serum (mMol/L). The total antioxidant status (TAS) parameter was evaluated using a Randox Total Antioxidant Status assay (SKU: NX2332) and glutathione peroxidase (GSH-Px) activity was measured using a Randox RANSEL Glutathione Peroxidase assay KIT (SKU: RS 505). The protocols in two replicates were performed with an ABX Pentra 400 biochemical analyzer (Horriba, France). The results were expressed as activity of GSH-Px in log10 U/L or as mMol/L for TAS.

### TBARS index in liver and feed samples

The concentration of thiobarbituric acid reactive substances (TBARS) in liver samples were determined using forced chemical oxidation induced by iron trichloride and sodium ascorbate^45^, after different incubation times (0, 60 and 120 min) at 37 °C. The measurements, in triplicates were performed using a Halo DB-20 double beam spectrophotometer (Dynamica Ltd., United Kingdom). The final TBARS concentration was expressed as mean ± standard deviation (SD) and given in nMoles of malondialdehyde (MDA) and in nMol/g of tissue.

### Antioxidant enzyme activity in pectoral muscle and liver tissues

The activity of catalase (CAT) and superoxide dismutase (SOD) was measured in the liver and pectoral muscles of the chickens. A 100 mg portion of each tissue was homogenized (Ultra-Turrax T25, Janke & Kunkel GmbH & Co. KG, Germany) in 0.8 mL ice-cold sucrose buffer containing 0.05 M Tris–HCl and 1 mM EDTA (pH 7.4). CAT activity, monitored as the decrease in H_2_O_2_ concentration at 25 °C^46^, was measured at 240 nm using the Halo DB-20 Double Beam Spectrophotometer. SOD activity, assessed at 450 nm on an microplate reader (EPOCH2) was measured as the inhibition of the xanthine/xanthine oxidase-mediated oxidation of cytochrome-c, utilizing a dedicated kit (19,160 SOD; Sigma Aldrich, St. Louis, MO, USA) with SOD from bovine erythrocytes used as a standard (S7571-15KU; Sigma Aldrich, Germany). The enzyme activities were expressed as units per L of supernatant for CAT, or as units per well for SOD. All analyses were performed in triplicates.

### Statistical analyses

All the measured variables were tested for normal distribution of the data (Shapiro–Wilk, *P* > 0.05). For normally distributed variables, a two-way ANOVA with the experimental factors of Oil, Q and Oil × Q interaction was conducted. Differences between parameters were tested according to the following statistical model (1):

1 $${\text{yijk}} =\upmu + \upalpha {\text{i}} +\upbeta {\text{j}} + \left( {\upalpha \upbeta } \right){\text{ij}} + {\text{eijk}}$$

where yijk—an observed value; µ—mean value of the population; αi—effect of the used oil; βj—effect of addition the optimized quercetin; (αβ)ij—interaction effect; eijk—effect of specific factors.

Differences between the means were determined according to a Tukey’s Multiple Comparison Test. In case of non-normality, a log transformation of the data was performed, and normality of the distribution was checked again. If normality was not confirmed, a nonparametric Mann–Whitney U test was used separately for the Oil and Q factors^47^. The TBARS in liver samples dataset was analyzed using a two-way repeated measures ANOVA, General Linear Model (GLM). A value of *P* ≤ 0.05 was considered significant and 0.05 < * P* ≤ 0.10 was discussed as a trend. All analyses were conducted using Statistica v.13.3 Software^48^. The tables show arithmetic means and standard error of the mean (SEM).

### Ethical approval

According to Polish law (Act of 15.01.2015 on the Protection of Animals Used for Scientific and Educational Purposes), the ethical approval for this experiment was not required because the experiment was carried out under standard production conditions, therefore, the birds were not exposed to excessive pain, suffering and stress. The experimental protocol was consulted and approved by the Advisory Team for the Welfare of Animals of The Faculty of Biology and Animal Science of Wrocław University of Environmental and Life Sciences (Decision no. 1/2020).

## Results

### Performance indices of broiler chickens

Selected performance parameters of broiler chickens are summarized in Table 2. The inclusion of FLO in the broiler diet had no effect on body weight gain (BWG) of chickens during the starter (day 1–14) and finisher (day 29–35) period, nor on the final body weight (day 35th). There was a trend (*P* = 0.07) towards a lower BWG in chickens fed with a FLO diet during the grower period, but the total BWG (day 1–35) of chickens was not affected. Dietary Q tended to improve the total BWG of chickens between day 1 and 35. The final body weight also tended to be higher (*P* = 0.10) in broilers fed with diets containing the optimized Q. There was no effect of oils and an optimized Q on the FCR during the grower or finisher feeding period. The lowered FCR in the starter period induced by dietary FLO (*P* = 0.03) tended also to be decreased by dietary Q (*P* = 0.08). However, the interaction of Oil × Q during the starter period was significant (*P* = 0.024) and have unfolded that the optimized quercetin prevented further decrease in the FCR in the chickens fed with the diet containing flaxseed oil (in FLO_Q group), but in turn, it decreased the FCR in chickens fed a diet supplemented with rapeseed oil (in RO_Q group).

**Table 2:** Average BWG and FCR of broiler chickens at the end of the starter, grower, and finisher period.

Items	Treatment				SEM	*P* value		
	RO	FLO	RO_Q	FLO_Q		Oil	Q	Oil × Q
BWG								
Starter [g]	378	409	411	406	7.2	0.54	0.84	N.A
Grower [g]	1200	1131	1243	1151	21.6	0.07	0.46	0.79
Finisher [g]	631	706	684	750	21.9	0.12	0.27	0.93
Total, d 1**–**d 35 [g]	2209	2246	2338	2308	27.5	0.94	0.10	0.55
*Final BW d 35 [g]	2256	2294	2384	2354	27.4	0.95	0.10	0.54
FCR								
Starter [kg]	1.49^a^	1.22^b^	1.25^b^	1.26^b^	0.03	0.03	0.08	0.02
Grower [kg]	1.51	1.56	1.51	1.58	0.02	0.17	0.86	0.92
Finisher [kg]	1.73	1.54	1.69	1.53	0.06	0.80	0.75	N.A
Total, d 1**–**d 35 [kg]	1.56	1.49	1.50	1.50	0.02	0.89	0.89	N.A

RO, diets with rapeseed oil (RO); RO_Q, diets with RO supplemented with the optimized dose of quercetin (Q); FLO, diets with flaxseed oil (FLO); FLO_Q, diets with FLO supplemented with the optimized dose of Q; BWG, body weight gain; FCR, feed conversion ratio; N.A.**—**not available**—**in the absence of normal distribution of the means**—**the Mann–Whitney U test was used to determine the effect of the oil and quercetin (Q) factor. *Final BW d 35: these data are included in supplementary material of a previous paper^33^. N.A.**—**not available**—**in the absence of normal distribution of the means (the Mann–Whitney U test was used for determining the effect of the Oil and Q factor). Letters (a,b) were added in case of interaction (Oil × Q; *P* ≤ 0.05) between oil and quercetin, and means sharing a common letter did not differ. Means without a letter did not differ (in the case of lack of interaction, at *P* > 0.05); SEM: standard error of the mean.

### Antioxidant status in blood and oxidative enzyme activity in liver and pectoral muscle

The total antioxidant activity of plasma, as estimated by ferric reducing antioxidant ability of plasma (FRAP), as well as the total antioxidant status (TAS) and an ability to neutralize free radicals (ABTS^·+^, DPPH^·^) did not differ between chickens fed with diets containing RO or FLO, nor the diets with optimized quercetin (Table 3). The type of oil (RO or FLO) and addition of Q also had no effect on antiradical activity (DPPH^·^) determined in the blood serum, nor on the activity of the GSH-Px measured in the whole blood. Chickens fed with diets enriched with FLO displayed higher CAT activity in liver tissue when compared to RO (*P* ≤ 0.05), but no significant effect of the optimized Q was confirmed. The Oil × Q interaction revealed a modulating effect of the optimized Q on liver CAT activity within the used oil (*P* = 0.03). This activity of liver CAT was slightly decreased in the FLO_Q group and increased in the RO_Q treatment, when compared to FLO and RO groups, respectively. At the same time, the level of liver CAT activity was maintained on the similar level between the FLO_Q and RO_Q groups. Liver SOD activity was not changed by the diets. Similarly, CAT and SOD antioxidant enzyme activity did not differ in the pectoral muscles of chickens. There was also no effect of dietary factors on the weight of chicken livers.

**Table 3:** Effects of oils and optimized quercetin on redox status in plasma and serum, the antioxidant enzymes in tissues of chickens, and the weight of the liver.

Items	Treatment				SEM	*P* value		
	RO	FLO	RO_Q	FLO_Q		Oil	Q	Oil × Q
Blood								
GSH-Px, whole blood [log10; U/L]	4.54	4.55	4.52	4.58	0.01	0.16	0.94	0.27
TAS, plasma [mMol/L]	2.09	2.05	2.12	2.05	0.03	0.59	0.86	N.A
FRAP, plasma [mMol/L]	0.61	0.60	0.60	0.67	0.03	0.98	0.72	N.A
ABTS, plasma [mMol/L]	1.76	1.78	1.73	1.77	0.03	0.66	0.74	0.84
DPPH, plasma [mMol/L]	0.36	0.37	0.38	0.39	0.01	0.60	0.45	0.99
DPPH, serum [mMol/L]	0.41	0.41	0.39	0.40	0.01	0.85	0.60	0.89
Liver								
CAT [U/mL]	6912^a^	9058^b^	8186^ab^	8220^ab^	247.42	0.02	0.64	0.03
SOD [U/well]	872.5	937.7	902.3	836.1	40.29	0.64	0.36	N.A
Pectoral muscle								
CAT [U/mL]	79.5	83.0	73.4	67.1	3.92	0.57	0.22	N.A
SOD [U/well]	54.6	53.0	52.6	50.8	2.49	0.60	0.60	N.A
Liver weight [g]	46.4	44.9	45.8	46.5	1.04	0.86	0.81	0.59

RO, diets with rapeseed oil (RO); RO_Q, diets with RO supplemented with the optimized dose of quercetin (Q); FLO, diets with flaxseed oil (FLO); FLO_Q, diets with FLO supplemented with the optimized dose of Q; N.A.**—**not available**—**in the absence of normal distribution of the means (the Mann–Whitney U test was used for determining the effect of the Oil and Q factor). Letters (a,b) were added in case of interaction (Oil × Q; *P* ≤ 0.05) between oil and quercetin. Means without a letter did not differ (in the case of lack of interaction, at *P* > 0.05); SEM, standard error of the mean.

### Oxidized lipid products in the liver

**Figure 1:**
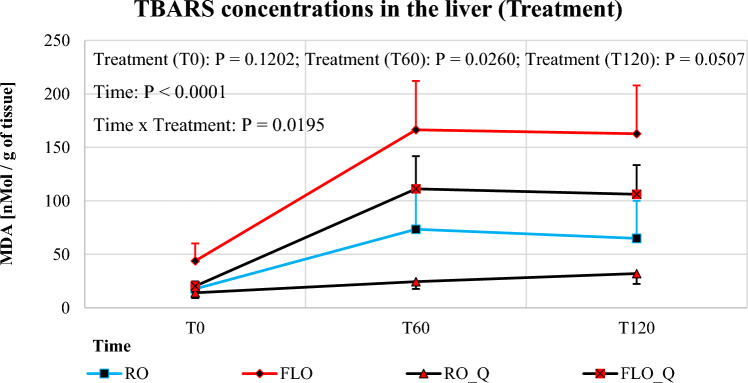
Effects of dietary treatments on lipid by-products in the liver. RO, diets with rapeseed oil (RO); RO_Q, diets with RO supplemented with the optimized dose of quercetin (Q); FLO, diets with flaxseed oil (FLO); FLO_Q, diets with FLO supplemented with the optimized dose of Q; T0, T60 and T120, time of sample incubation during oxidation in iron trichloride and sodium ascorbate. N.S.—not significant (*P* ≥ 0.05); Error bars represent standard deviation of the mean. The thiobarbituric reactive substances (TBARS) in the liver were measured at different time points (Time) during forced chemical oxidation. Effects of treatment was tested by repeated measures of the TBARS data along the kinetics (T0, T60 or T120 min).

During the samples were not subjected to forced oxidation, no differences in the levels of liver TBARS were noticed between the four treatment groups (mean ± SD: RO = 18 ± 5; FLO = 44 ± 16; RO_Q = 14 ± 5; FLO_Q = 20 ± 4 nMol/g of tissue) (Fig. 1). After 60 min of the reaction, FLO livers exhibited higher TBARS concentrations (166 ± 46 nMol/g of tissue; *P* = 0.0260) in comparison with the livers obtained from the RO_Q group (24 ± 7 nMol/g of tissue). This trend was maintained after 120 min of sample incubation (FLO = 163 ± 45; RO_Q = 32 ± 10 nMol/g of tissue; *P* = 0.0507). During the comparing liver tissue obtained from the FLO (163 ± 45 nMol/g of tissue) and FLO_Q groups (111 ± 31 nMol/g of tissue) to that of the RO (73 ± 37 nMol/g of tissue) and RO_Q treatments (24 ± 7 nMol/g of tissue), we observed a significant increase in the TBARS index, especially between timepoints T = 0 and T = 60 min of peroxidation. No significant increase in liver MDA was seen after 120 min of oxidation (RO = 65 ± 35, FLO = 163 ± 45, RO_Q = 32 ± 10, FLO_Q = 106 ± 27 nMol/g of tissue).

**Figure 2:**
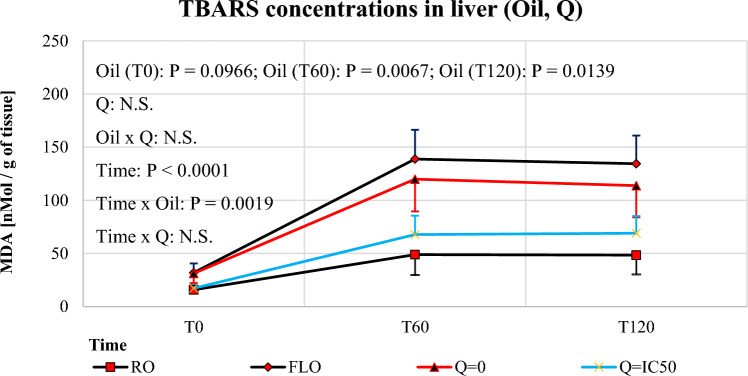
Effects of oils and optimized quercetin on lipid by-products in the liver. RO, diets with rapeseed oil (RO) and diets containing optimized dose of Q (RO_Q); FLO, diets with flaxseed oil (FLO) and diets with optimized dose of Q (FLO_Q); Q = 0, diets with RO or FLO without the optimized dose of Q (RO and FLO); Q = IC50, diets with RO or FLO supplemented with the optimized dose of Q (RO_Q and FLO_Q); T0, T60 and T120, time of sample incubation during oxidation in iron trichloride and sodium ascorbate; N.S.**—**not significant (*P* ≥ 0.05); Error bars represent standard deviation of the mean. The thiobarbituric reactive substances (TBARS) in the liver were measured at different time points (Time) during forced chemical oxidation (T0, T60 or T120 min). The effects of oil (Oil), quercetin (Q) and their interaction (Oil × Q) were tested by repeated measures of the TBARS data along the kinetics. Oil (T0): *P* = 0.0966: effect of oil factor showed a trend for increscent of TBARS values in liver samples at time-point = 0 min. Oil (T60) and Oil (T120): effect of oil factor on TBARS values was significant a time-point 60 (*P* = 0.0067) and 120 min (*P* = 0.0139).

Dietary FLO tended to increase liver TBARS values (*P* = 0.0966) at start of incubation (FLO = 32 ± 9 nMol/g of tissue; RO = 16 ± 3 nMol/g of tissue), and increased the TBARS index in the FLO liver samples (*P* = 0.0067) after forced oxidation for 60 min (FLO = 139 ± 27 nMol/g of tissue; for RO = 49 ± 19 nMol/g of tissue) and 120 min (FLO = 134 ± 26 nMol/g of tissue; for RO = 48 ± 18 nMol/g of tissue; *P* = 0.0139) (Fig. 2).

By putting together results from liver TBARS index of unsupplemented chickens (Fig. 2, marked as ‘Q0’ for RO and FLO groups) to the ones from the optimized quercetin diet (Fig. 2, marked as ‘Q = IC50’ for RO_Q and FLO_Q groups), no significant difference was seen. At the incubation time points = 0, 60 and 120 min, the TBARS concentrations in the ‘Q0’ samples were as follow: 31 ± 9, 120 ± 30 and 114 ± 30 nMol/g of tissue, respectively. For the ‘Q = IC50’ chicken livers, the average MDA concentration after 0, 60 and 120 min of oxidation was: 17 ± 3, 68 ± 18 and 60 ± 16 nMol/g of tissue, respectively. A time-dependent increase of TBARS was confirmed for all chicken liver samples during the incubation period (*P* < 0.0001). This trend was altered by the oil used in the feed (Time × Oil; *P* = 0.0019), but not affected by optimized quercetin (Time × Q; *P* ≥ 0.05).

## Discussion

The addition of fat to the feed of poultry is a common practice essential to meet the required amount of energy, as well as it improves the absorption of fat-soluble vitamins, the structure of the feed and its palatability. Compared to feeds with similar nutritional value, chickens fed oil-containing diets perform better than birds fed oil-free diets^49^. The application of dietary oils rich in n − 3 FA may also be a useful method for modulation of fatty acid composition in the chicken meat to improve its nutritional value^33^.

Inclusion of flaxseed oil (FO) in chicken diets had no influence on the final BWG of broilers, nor on other performance traits related to feed intake during the grower, finisher, and total period of the experiment. Our results are in accordance with previous findings^50–52^, as the replacement of linseed oil with other types of oils (i.e., sunflower, soybean, or rapeseed oil) did not have any effect on the final performance of broilers. As shown by Mandal et al*.*^53^, even after a radical change in the proportions of omega-6/omega-3 was induced by dietary linseed oil (i.e., from 21.55–25–33 to 5.43–5.95), broiler chicken performance was not improved. What is more in other studies the incorporation of omega-3 FA to the broiler’s diet was linked to an overall beneficial effect on bird welfare and has been shown to improve the immunity^54,55^, liver functions^54^ and serum lipid profile of broiler chickens^56^. Moreover, a higher proportion of dietary linolenic acid (ALA) facilitates its conversion into the active metabolites such as ‘eicosapentaenoic acid’ (EPA) and ‘docosahexaenoic acid’ (DHA). The presence of this compounds lowers the inflammatory response and is critical for the optimal functioning and structure of cell membranes, tissues, and vital organs^57^. By increasing local oxidative stress within the lumen of the chicken gut, diets rich in omega-3 have also been shown to reduce cecal coccidiosis induced by *Eimeria tenella*^58^.

In this study, inclusion of optimized concentrations of quercetin tended to improve the total BWG and the final BW of chickens. However, irrespective of the source of dietary fat, optimized Q did not change the total feed conversion (day 1–d 35), although the significant interaction (Oil × Q) was observed for the starter period of the experiment. The present results are in accordance with the findings of Zhang and Kim^59^, who reported an improved final BWG, with a linear trend and a quadratic effect of dietary Q between the concentration 250 and 500 mg/kg. In contrast, Goliomytis et al*.*^3^ reported no effect of dietary Q on the final BW of broilers (day 42), and a significant increase in the FCR of chickens after higher concentration of this flavonol (i.e.: 0.5 and 1.0 g/kg of feed) was incorporated in the diet. Meanwhile, Zhang and Kim^59^ showed that increased level of tumor necrosis factor α (TNF-α) may indicate the ability of dietary quercetin to enhance the immune capacity of broiler chickens. Furthermore, bacteriostatic properties favoring the development of intestinal populations of ileal *Lactobacillus* spp. was detected^59^. In our previous report from this project^33^ we manage to show that optimized quercetin improved significantly the oxidative stability of broiler chicken meat obtained from chickens fed with FLO_Q and containing higher proportion of n − 3 fatty acids (FA) diet. The current results, supplementary to the previous study, provide compelling evidence for the possibility of developing optimized antioxidant feed additives based on polyphenol compounds, without any negative effects on the performance of chickens.

Our results concerning the mechanisms involved in maintaining redox status (FRAP/TAS, DPPH^·^/ABTS^·+^) and the activity of GSH-Px in the blood of chickens did not confirm any effect of dietary oils and optimized quercetin on the systemic antioxidant status. Dong et al.^60^ reported a decrease in the total antioxidant capacity (TAOAC) of the blood serum of chickens fed with a diet containing oxidized soybean oil. This was not alleviated by dietary quercetin used at 200, 400 and 800 ppm in the chicken diets. The same authors also confirmed no effect of dietary Q on blood serum GSH-Px on the 18th and 42nd day of life, but GSH-Px activity in the 800-ppm treatment was higher on day 11. When up to approx. 6 g of dietary quercetin per kg was added to chicken diet, Rupasinghe et al*.*^2^ found the total antioxidant capacity (TAC) in the plasma and liver measured by FRAP was unchanged. This could be related to other dietary antioxidants present in the feed, including vitamin E, ethoxyquin, and the antioxidant present in grain. This insight may also be relevant to the present results, since antioxidants ingredients such as α-tocopherol or butylated hydroxyanisol were a part of the added mineral-vitamin premix (Table 1) and could have masked the final effect of quercetin in both experiments.

Total antioxidant status considers collective reactions: both between low molecular defenses (α-tocopherol, ascorbate, carotenoids) and endogenic antioxidants typically not provided with diet (i.e., glutathione, uric acid, melatonin, etc.)^61^. Thus, TAS reveals the total systemic redox status of the organism. This parameter seems to be prone to changes by complex factors linked to an increased formation of reactive oxygen species (ROS) and oxidative stress, including sanitary^62^ or heat stress^63^. In pigs subjected to 6-weeks of sanitary stress, an increased response in the activity of oxidative enzymes (SOD, CAT, glutathione reductase—GSH-Rx) was observed in the liver and perirenal fat. Nonetheless, the systemic redox status was altered and displayed significantly increased serum diacron reactive oxygen metabolites (dROM), decreased plasma FRAP, and a decrease in the body weight of pigs. This indicates the inadequacy of the systemic antioxidant defense system, despite its increased activation^62^.

Stress conditions were not applied in the present study, however, the diets containing flaxseed oil could act as a potential dietary stress factor. Due to the fact that FLO has higher potential to oxidate and low stability it can contribute with increasing pool of dietary oxidized fats^33^, what leads to increasing formation of ROS and oxidative stress in chicken^64^. In this work, the average level of MDA determined in starter, grower, and finisher FLO and FLO_Q diets was approx. 4- and 1.7-fold higher (50 ± 17 and 29 ± 5 nMol/g of feed, respectively) than in RO diets (12 ± 4 nMol/g of feed). This was reflected by the increased TBARS index (*P* ≤ 0.05) found in the unincubated (i.e., not subjected to forced oxidation) livers obtained from the FLO and FLO_Q chickens, and was in accordance with the increased liver CAT activity observed in the FLO chickens. Studies have confirmed that chickens fed with diets containing increased amounts of oxidized fats have decreased body weight gain^65^ and higher MDA levels in their meat^66^, blood plasma^67^ and livers^68^. As a crucial organ involved in the metabolism of nutrients and detoxifying processes^60^, the liver must cope with the increased formation of ROS and any oxidative stress related with nutrition. Long et al*.*^69^ described the decreased activity of liver CAT, SOD, and glutathione reductase (GSH-Rx), decreased vitamin E levels, and increased MDA content (*P* < 0.05) after the incorporation of oxidized fish oil into a ‘hybrid grouper’ fish diet (*Epinephelus fuscoguttatus* × *E. lanceolatus*), in a dose dependent manner. In broiler chickens, the peroxidability of the liver may be enhanced by oils in the diet that have a higher content of polyunsaturated fatty acids (PUFA) and a lower share of monounsaturated fatty acids (MUFA)^70^, further deposited in this tissue^71^. In the present study, the proportion of dietary α-linolenic acid (ALA, C18:3, n − 3 PUFA) was increased by about 8.25–7.56-fold in the FLO and FLO_Q diets, while the content of oleic acid (C18:1, MUFA) was reduced by about 2.18–2.5-fold, relative to the RO and RO_Q feeds (Table 1). Considering the approx. 25–100-fold increase in peroxidability of ALA in relation to oleic acid (C18:1)^72,73^, the time-depended increase of TBARS concentrations in the FLO liver samples (FLO and FLO_Q group) after 60 and 120 min of forced oxidation, could be related to the increased deposition of this fatty acid in the liver of FLO and FLO_Q chickens. On the other hand, linoleic acid (LA, C18:2, n-6 PUFA) which is characterized by approx. 10–40-fold lower peroxidation stability than oleic acid, was present in all the diets in an unchanged proportion. This suggests that it could have had a comparable effect on chemically induced peroxidation in the liver of chickens from all the groups.

For the neutralization of ROS, organisms use enzymatic responses. These enzymes include SOD, glutathione peroxidase (GSH-Px) and reductase (GSH-Rx), and CAT. CAT enzyme belongs to the oxidoreductases group and decomposes hydrogen peroxide into water and oxygen in a disproportionation reaction. The increase in CAT activity during inflammatory processes seems to be a defensive reaction of the organism^74^, but enzyme activity may decrease with a prolonged exposure to oxidative stress^75^. In our study, the optimized Q did not restore liver CAT activity of FLO_Q chickens to the levels observed in the livers of RO diet chickens (*P* > 0.05). Despite a similar bioavailability of quercetin in avian species and in mammals, the possibility of a deeper deposition of sulfate, glucuronide, glucoside or isorhamnetin glucoside in the liver tissue might not be sufficient to affect the liver FRAP values^2^. Otherwise, the insufficient protective effect of dietary Q may also be related to the lower antioxidant activity and TEAC values (FRAP, ABTS^·+^) exerted by Q’s metabolites in general (*P* ≤ 0.05), as confirmed for different phenolic compounds that may be produced in the colonic degradation^76^. Interestingly, our results revealed a significant interaction between Oil × Q factors, indicating a possible modulation of liver CAT activity induced by the optimized Q used in this study. Hence, it is possible that optimized Q could reduce the liver burden in the FLO_Q chickens by decreasing the by-products of peroxidation provided with the diet, up to 75.9%, in relation to FLO group^33^. But, when the diet was supplemented with optimized Q in combination with relatively stable rapeseed oil (in RO_Q group), it contributes to the improvement of liver CAT activity to the level observed in the livers obtained from the FLO_Q chickens.

By transforming the superoxide radical into hydrogen peroxide and oxygen in a dismutation reaction, superoxide dismutase (SOD) is the first line of enzymatic defense against increased formation of ROS^74^. A decrease in liver SOD activity may be one of the indicators of peroxyl radical accumulation and may coexist with the increased levels of MDA^77^. In the present study, liver SOD was not affected by the plant oils and Q, which was observed also in the unchanged activity of SOD and CAT in pectoral muscle within all groups. After changing the proportion of linseed oil in the diet of chickens from 2 to 4%, El-Bahr et al.^78^ reported a decrease in liver SOD and oxidized and reduced glutathione (GSSG, GSH), as well as an increase in MDA and deoxyguanosine oxidative damage (8-OHdG). However, the improvement in performance of chickens receiving the 4% linseed diet (*P* ≤ 0.05), including higher BWG, lower feed intake, and unaffected FCR, may exclude the occurrence of chronic oxidative stress in the cited study. The reduced BWG and feed intake associated with altered total antioxidant capacity and lower activities of SOD and CAT in the blood of chickens after incorporating 4% oxidized oil to the diet, was demonstrated previously^79^.

From the literature, it seems that dietary polyphenols are more effective in improving antioxidant parameters under stress conditions, than at typical experimental conditions. Choi et al*.*^80^ reported that administering quercetin to mice at 100 or 250 mg/kg BW for 18 days had no effect on liver SOD under normal conditions (control group) but restored the SOD levels in mice that were subjected to oxidative stress induced by 7,12-dimethylbenz(a)anthracene (DMBA). The results of Dong et al*.*^60^ showed increased activity of liver SOD in chickens fed with diets containing oxidized fat, while 200 ppm of dietary Q displayed an opposite effect. The cited authors indicated no effect of oxidized oil and Q on the relative weight of the liver, which is similar to the results in the present study. A Q-induced linear increase of SOD in the pectoral muscles of chickens was confirmed by Zhang and Kim^59^. This stands in contrast to our data, but in their study the authors applied quercetin in much higher concentrations (250, 500 and 1200 mg/kg) and used it in feed mixtures containing soy oil—a plant oil that is even more stable in terms of peroxidation than the flaxseed oil used in our work.

It is important to note that the regulation of antioxidant responses in the presence of polyphenols is multipath and still not fully determined. In the case of quercetin, factors include the direct effects of Q on signal transduction pathways, dose-dependent impact on GSH content, decreasing the environmental and toxicological dependent level of ROS, modulating activity of antioxidant enzymes^81^, the inhibition of the xanthine oxidase superoxide anion, as well as quenching singlet oxygen and hydroxyl radicals^82^. In human hepatoma cells, quercetin activated its protective mechanisms through mRNA levels of glutamyl cysteine-synthetase (GCS), through expression levels and activity of GSH-Px and GSH-Rx, and through improved glutathione content after 4 h of incubation^83^. By upregulating the transcription of the nuclear factor erythroid 2-related factor (Nrf2) and its downstream genes, and by inducing a higher expression of mucin 2 (MUC2), quercetin was able to restore redox balance and reinforced the intestinal barrier in broiler chickens fed with diet containing oxidized oil^54^. The response of the organism may be tissue-specific, while also depending on dose and on the species.

## Conclusions

Application of flaxseed oil to chicken diets did not change the global performance traces of the chickens, but the total BWG and final BW tended to be improved by incorporation of the optimized quercetin. While quercetin seemed to decrease the negative effect of oxidized fats on liver CAT activity, finding other antioxidant and performance traces unaltered indicated the lack of systemic oxidative stress within all dietary treatments. The results indicate that, when based on the peroxidability of the used fat source, optimizing the addition of natural polyphenols may be a valuable alternative to the application of the polyphenolic antioxidants in animal nutrition. The more conscious and targeted utilization of polyphenolic or other antioxidant substances as customized to the peroxidability of fat sources could be useful for their more economical and safe use in livestock nutrition, contributing the conception of sustainable development, covering ‘The European Green Deal’^84^ and ‘Farm to Fork Strategy’^85^, aiming to reduce the environmental and climate impact of agricultural production whilst ensuring equitable economic returns for the farmers.

## Data Availability

The data used in this study are available from the corresponding author on reasonable request.
